# Sex differences in severity and mortality from COVID-19: are males more vulnerable?

**DOI:** 10.1186/s13293-020-00330-7

**Published:** 2020-09-18

**Authors:** Ajay Pradhan, Per-Erik Olsson

**Affiliations:** Biology, The Life Science Center, School of Science and Technology, Örebro University, SE-701 82 Örebro, Sweden

**Keywords:** Coronavirus, Immune system, Gender, Sex hormones, Pathogenesis

## Abstract

Coronavirus disease 2019 (COVID-19) has shown high infection and mortality rates all over the world, and despite the global efforts, there is so far no specific therapy available for COVID-19. Interestingly, while the severity and mortality of COVID-19 are higher in males than in females, the underlying molecular mechanisms are unclear. In this review, we explore sex-related differences that may be contributing factors to the observed male-biased mortality from COVID-19. Males are considered the weaker sex in aspects related to endurance and infection control. Studies show that viral RNA clearance is delayed in males with COVID-19. A recent study has indicated that the testis can harbor coronavirus, and consequently, males show delayed viral clearance. However, the role of testis involvement in COVID-19 severity and mortality needs further research. Males and females show a distinct difference in immune system responses with females eliciting stronger immune responses to pathogens. This difference in immune system responses may be a major contributing factor to viral load, disease severity, and mortality. In addition, differences in sex hormone milieus could also be a determinant of viral infections as estrogen has immunoenhancing effects while testosterone has immunosuppressive effects. The sex-specific severity of COVID-19 infections indicates that further research on understanding the sex differences is needed. Inclusion of both males and females in basic research and clinical trials is required to provide critical information on sex-related differences that may help to better understand disease outcome and therapy.

## Introduction

In December 2019, a new pneumonia outbreak emerged in Wuhan, Hubei province, China, which showed high infection rate and mortality. Similar to severe acute respiratory syndrome coronavirus (SARS-CoV), patients displayed symptoms of pneumonia [1, 2]. Later, the Chinese Center for Disease Control and Prevention (CDC) identified that this disease was caused by coronavirus and it was classified as 2019 novel coronavirus (2019-nCoV), and the disease was named coronavirus disease 2019 (COVID-2019). The virus was renamed by the International Committee on Taxonomy of Viruses as severe acute respiratory syndrome coronavirus-2 (SARS-CoV-2) [3]. The World Health Organization (WHO) declared SARS-CoV-2 a pandemic on 11 March 2020 [3]. The origin of this coronavirus is not yet known; however, it is speculated to originate from a live animal market in the Hubei province, China [1].

This is the third coronavirus outbreak in the past few years. In 2002–2003, a coronavirus (SARS-CoV) emerged in China and it resulted in 774 deaths. In 2012, Middle East respiratory syndrome (MERS) which first emerged in Saudi Arabia resulted in 858 deaths [4].

The SARS-CoV-2 is mainly transmitted from person-to-person via respiratory droplets, and the clinical features of COVID-19 range from asymptomatic respiratory infections to severe pneumonia [5]. The most common symptoms are fever, fatigue, dry cough, and myalgia [4]. Older patients (≥ 65 years) show severe symptoms of COVID-19, and among them, more men develop serious symptoms and show higher mortality compared with women [6, 7]. Patients with underlying comorbidities including diabetes, hypertension, and cardiovascular disease in both young and older individuals show more severe symptoms and higher mortality [2, 6].

**Fig. 1:**
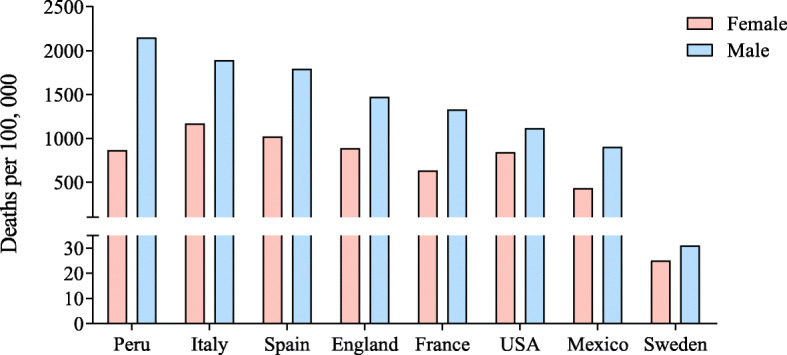
Sex-biased mortality from COVID-19. The data from different countries show that male mortality is higher than females. The data for Peru, Italy, Spain, England, France, USA, and Mexico was obtained from Global Health 50/50 which was updated July 12. The mortality data as of July 24 for Sweden was taken from the Swedish Public Health Agency

To date, there is no specific therapy to treat SARS-CoV-2 infection. Different drugs that were designed for other diseases including HIV, Ebola, and malaria have been tested to treat COVID-19 [3]. The lack of vaccines and other specific drugs against COVID-19 has contributed to the high mortality. As of July 28, 2020, according to John Hopkins University database, 188 countries have shown infections; 16,481,230 confirmed cases and 654,052 mortality have been recorded. Interestingly, COVID-19 has shown clear sex-specific mortality with higher death rate in males compared with females (Fig. 1). An analysis of COVID-19 data from the other 29 countries that were not included in this study also showed male-biased mortality [8]. The confirmed cases of SARS-CoV-2 infections differed across the age groups between males and females. While females in the age group 10 to 50 years showed higher incidence, males before 10 and after 50 years were more susceptible [9]. Analysis of viral RNA in COVID-19 patients indicated that the males show delayed viral clearance as the SARS-CoV-2 RNA was detected for a longer time in males compared with females [10, 11]. However, there is no clear understanding of why males are showing higher mortality compared with females. Hence, this review will highlight different theories/sex differences to understand the COVID-19 male-biased death rate. Understanding of sex differences could further help in understanding the disease outcome and therapeutics for COVID-19 and other new emerging diseases.

## Coronavirus infection and therapeutic targets

**Fig. 2:**
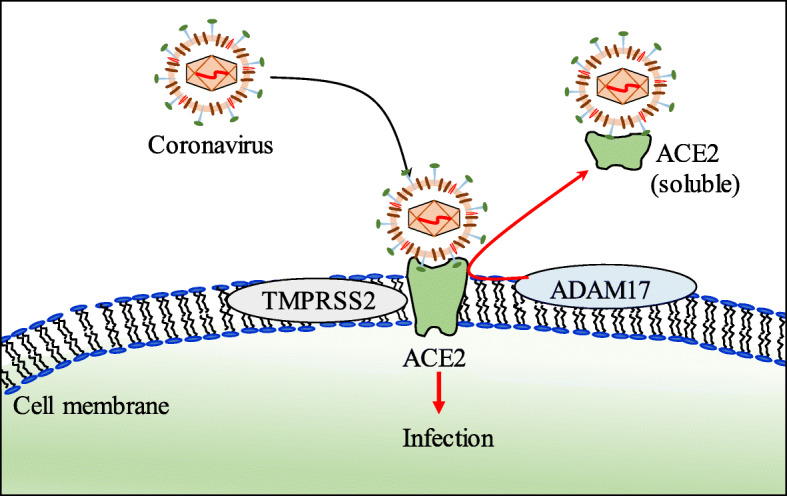
Coronavirus entry into cell. Coronavirus spike protein binds to ACE2 on the human cell surface. TMPRSS2 cleaves spike protein to help virus fusion and entry inside the target cell. ADAM17 can cleave ACE2 and release the ectodomain as a soluble form. The soluble form can bind to virus and can help to prevent infection

**Fig. 3:**
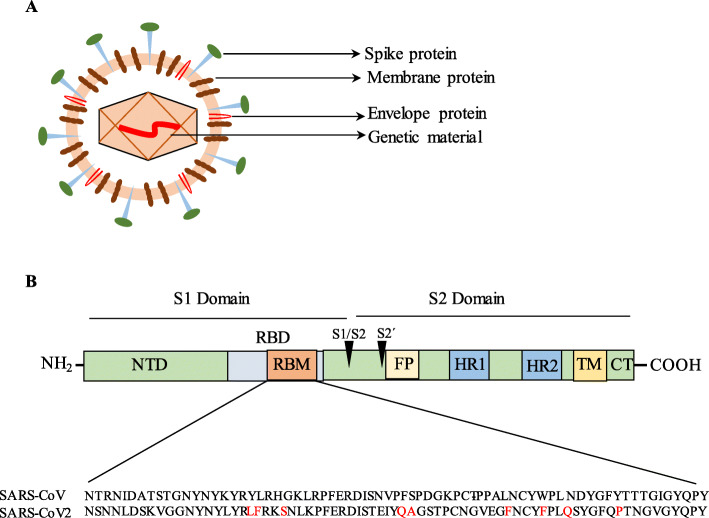
Spike protein of coronavirus. The schematic diagram shows the coronavirus structure (**a**). The spike protein (S) has S1 and S2 domains. The S protein has receptor binding domain (RBD) and the receptor binding motif (RBM) and within RBD binds ACE2. S1/S2 and S2′cleavage sites are acted by protease to assist viral fusion. RBM sequence between SARS-CoV and SARS-CoV-2 showed 46.5% identity, and the nine residues that increase RBM affinity to ACE2 are indicated in red (**b**). NTD, N-terminal domain; RBD, receptor binding domain; RBM, receptor binding motif; FP, fusion protein; HR1, heptad repeat 1; HR2, heptad repeat 2; TM, transmembrane domain; CT, cytoplasmic tail

Coronaviruses are pleomorphic, enveloped, single positive stranded RNA virus [12]. The viral membrane contains transmembrane (M) glycoprotein, spike (S) glycoprotein, and envelope (E) protein [13]. The spike protein forms the coronal fringe around the virus, and it facilitates viral entry into human cells [13, 14] (Fig. 2). The S protein contains the S1 and S2 subunits where the S1 domain is linked to receptor binding and the S2 domain is required for cell membrane fusion. The S1 subunit of the S protein contains the N-terminal domain (NTD) and a receptor binding domain (RBD) [3] (Fig. 3). The RBD binds to angiotensin-converting enzyme 2 (ACE2) and uses it as the entry receptor [14–16]. Apart from ACE2, the human serine protease, transmembrane protease/serine subfamily member 2 (TMPRSS2), is also critical for virus entry into the host cell as it is involved in S protein priming [14, 17, 18]. Serine proteases on the surface of target cell cleave the S protein at the S1/S2, S2 cleavage site, and given its important role in viral entry, TMPRSS2 is considered a potential therapeutic target to control coronavirus infection [14]. TMPRSS2 inhibitors including nafamostat and camostat are undergoing clinical trials to determine their efficacy against COVID-19 [5].

The S2 domain contains fusion peptide (FP) and heptad repeat (HR) 1 and 2 [3] (Fig. 3). Following binding of RBD to ACE2, the S2 domain undergoes conformational changes to facilitate membrane fusion and these events facilitate virus entry into target cells [3]. The importance of S protein, especially the RBD region, makes it a potential target for SARS-CoV-2 vaccine and antibody-based drug development [19].

Analysis of RBD region of SARS-CoV and SARS-CoV-2 sequence using CLUSTLW showed that there is only 46.5% homology (Fig. 3). Despite the low sequence homology, the binding affinity of SARS-CoV-2 with ACE2 was found to be higher than SARS-CoV [20, 21]. Different research groups, using protein-protein interaction studies, have identified the binding mechanisms of the SARS-CoV-2 RBD to human ACE2 [20–24]. Nine residues (L455, F456, S459, Q474, A475, F486, F490, Q493, and P499) critical for RBD affinity to ACE2 have been identified [24]. When these nine residues were mutated and changed to those of the SARS-CoV sequence, reduced binding affinity was observed [24]. Out of these nine residues, six residues (L455, F456, A475, F486, F490, Q493) were also shown to be important by other groups [20–23]. Apart from these nine residues, another 12 residues (L417, G446, Y449, y453, N487, Y489, G496, Q498, T500, N501, G502, Y505) have been identified to be important for RBD contact with ACE2 [20–23]. These studies show that the SARS-CoV and SARS-CoV-2 binding mechanisms to the ACE2 receptor are similar. When the RBD from either SARS-CoV or SARS-CoV-2 was injected in mice, a strong clade-specific neutralizing antibody production was initiated. However, a weak cross-neutralizing activity was observed, which suggested that the RBDs of SARS-CoV and SARS-CoV-2 have unique antigenic features [24].

ACE2 is a membrane-bound transmembrane aminopeptidase that is expressed in different tissues including the heart, intestine, kidney, lungs, lymph nodes, and testis [25, 26]. In the lungs, ACE2 is mainly expressed in type I and type II alveolar epithelial cells [25]; however, its function in the lungs is not clear [27]. ACE2 undergoes post-translational modifications where a disintegrin and metalloproteinase 17 (ADAM17) cleaves ACE2 to release the ectodomain which has the catalytic domain and that can bind coronavirus [28] (Fig. 2). ACE2 also undergoes post-transcriptional modification where the *miR-421* binds to 3′-UTR of ACE2 and modulates its expression [29]. This suggests that apart from ACE2 expression, regulation of *miR-421*, TMPRSS2, and ADAM17 is important to understand COVID-19 pathogenesis.

Different ACE2 variants are reported that show sex-specific expression [30–32]. Since ACE2 is an X chromosome encoded gene, the presence of disease or risk variants in men will be more detrimental [31]. It is suggested that the missense ACE2 variants could influence binding with spike proteins and this, in turn, will affect the progression of COVID-19 [31]. However, in another study, the analysis of ACE2 variants in an Italian population indicated that there was no association with COVID-19 severity and sex-biased mortality [30]. A wider analysis of data from different countries may further help to know whether ACE2 variants can affect the COVID-19 pathogenesis.

In patients with heart diseases, the level of plasma ACE2 is high [33] but unable to provide protection against COVID-19 [5]. This suggests that ACE2 is important for SARS-CoV-2 cell entry but that its expression levels do not correlate with the degree of infection and viral load [34]. The equilibrium between soluble and membrane-bound ACE2 should also be analyzed as it might influence the COVID-19 pathogenesis [33].

The renin-angiotensin system (RAS) plays an important role in maintaining blood pressure, fluid homeostasis, and salt balance. ACE2 is a homologue of ACE, but they have opposite functions with ACE involved in activation of RAS by conversion of angiotensin I to angiotensin II. On the other hand, ACE2 negatively regulates RAS system by inactivating angiotensin II [35, 36]. The modulation of RAS system using angiotensin receptor blockers, angiotensin II, and ACE inhibitor is considered to be a potential therapeutic target against COVID-19 [5]. An in vitro study showed that human coronavirus (HCoV-NL63) infection can downregulate ACE2 [37] and similar findings were observed with SARS-CoV infections in mice model [38]. The reduced ACE2 levels following SARS-CoV infection led to acute lung injury, and interestingly, the lung injury was significantly attenuated in ACE2 knockout mice and angiotensin II type 1 receptor blocker (ARB) treated groups [38]. In animal models, while ACEI and ARB treatments increase ACE2 receptors, clinical data for similar effects are lacking [5, 39]. Based on this, it was argued that patients under ACEI and ARB medication may be at increased risk of severe outcomes from COVID-19 [34, 39]. The study by Sama et al. found that plasma ACE2 levels are high in men compared to women in patients with heart failure [33]. However, the use of ACE inhibitors and ARB was not associated with higher plasma ACE2 levels [33].

Recombinant human ACE2 (rhACE2) is being considered as a therapy to control SARS-CoV-2 infection. The idea is to use the soluble rhACE2 as a decoy receptor to bind SARS-CoV-2 and reduce the viral load and infection [5]. The rhACE2 was considered for SARS-CoV treatment [38], but clinical trials for rhACE2 showed fast clearance rates [40]. A pilot clinical trial (ClinicalTrials.gov #NCT04287686) of rhACE2 for treatment of COVID-19 was initiated, but this study was withdrawn on March 17, 2020 [5]. Another clinical trial (ClinicalTrials.gov #NCT04335136) is underway to analyze the efficacy of rhACE2 against COVID-19. The pharmacological problems with rhACE2 have led to another novel approach where immunoglobulin is combined with rhACE2 (extracellular domain) to generate a fusion protein. This approach has shown promising pharmacological properties in animal models and suggested to be a good system for diagnosis, prophylaxis, and treatment of SARS-CoV-2 [41].

## Are males more vulnerable to stress?

The high mortality of males due to COVID-19 raises the question whether males are more vulnerable than females. Males show higher mortality from diseases including heart disease, diabetes, liver disease, and cancer [42]. Since these diseases are known to show sex-specific occurrence [43, 44], they could be contributing factors for the sex-biased mortality from COVID-19. For instance, the male mortality in Italy was reported to be high compared to China and this is suggested to be due to a higher prevalence of cardiovascular diseases in Italian men [6]. The differences between males and females are observed not only in disease susceptibility but also in early development during infancy, endurance towards stress conditions, and overall life expectancy [45, 46]. Sex differences are observed in life expectancy, with females outliving males by almost 7 years in some countries [47]. This difference in life expectancy may be due to sex-related differences including hormonal milieu, genetics, and other physiological traits. Pieces of evidence indicate that females can outlive males even in harsh climate including famine [46]. The 1772–1773 famine in Sweden dropped the life expectancy of males to 17.15 years while it was 18.79 years for females. The Irish famine (1845–1849) dropped life expectancy to 18.7 years for men and 22.4 years for women. The Ukrainian famine in 1993 dropped the life expectancy of males from 41.58 to 7.3 years and 45.93 to 10.9 for females [46]. Other historical events also suggest that endurance levels to cope up with stressful conditions are low in males compared to females. The freed American slaves’ journey back to Africa in 1820–1843 resulted in high mortality with 43% death rate in the first year. The life expectancy at birth was 1.68 years for males and 2.23 years for females [46]. The measles outbreak in Iceland (1845–1849) also showed sex-specific mortality. The average life expectancy dropped from 37.62 years to 16.76 for males and 43.99 years to 18.83 years for females [46].

Sex ratio at birth is also sensitive to changes in the environment. For instance, natural calamity can lead to a decrease in the male birth rate. The Kobe earthquake in January 1995, where thousands of lives were lost and many thousands became homeless, correlated to low male birth rates during that year [48]. The terrorist attack of September 11, 2001, and the great recession of 2007 also resulted in decreased male childbirth [49, 50].

Taken together, it can be suggested that males have lower endurance levels than females. The sex-related difference in the immune system, sex hormone milieu, and other unknown causes may be a contributing factor for the high mortality of males in stressful conditions including COVID-19.

## Immune system differences and viral infection

Sex differences result in differential regulation of innate and adaptive immune response which in turn regulates sex-biased pathogenesis and mortality towards various pathogens [51, 52]. Males and females show differences in immune response when challenged with viral infections. For instance, in acute HIV infected cases, females show 40% less viral RNA than males, and hepatitis virus shows higher mortality in men [51]. Furthermore, there are many bacterial, fungal, and viral diseases that show sex-biased infections. *Treponema pallidum* (syphilis), *Borrelia burgdorferi* (Lyme disease), *Cryptococcus neoformans* (fungal meningitis), *influenza A* (influenza), and *hepatitis C* (hepatitis) infections are common in males while *Candida albicans* (onchomycosis), *Escherichia coli* (bacteremias), and *Taenia* (tapeworm) infections are common in females [52].

COVID-19 has also shown clear sex-specific bias with males showing severe response and higher mortality. This sex-biased mortality could be attributed to sex differences in immune response as females are known to mount more robust innate, cell-mediated and humoral immune response than males [51, 53, 54]. Although females mount higher immune response when infected with pathogens, the heightened immune response can also lead to immunopathology. Immune-related diseases show sex-specific incidences, for instance, most of the autoimmune diseases including rheumatoid arthritis, systemic lupus erythematosus, Sjögren syndrome, myasthenia gravis, and multiple sclerosis are prevalent in females [51, 55].

Females show higher antibody response to vaccines, and consequently, vaccine efficacy is high in females [56–58]. For instance, the protective antibody responses against the measles vaccine were twice as high in females compared to males of 11–22 years of age [58]. Similar effects have also been observed with other vaccines including rubella, hepatitis, rabies, mumps, yellow fever, and influenza [57]. Although females respond better to vaccination, they also show higher adverse reactions including pain, fever, and inflammation to vaccines [57]. Interestingly, in the early years (birth to 5 years), males are known to develop more robust immune responses than females [51]. This sex-specific differences in immune response in this early stage could be due to differences in immune cell types as young males are known to have a higher percentage of natural killer cells while females have higher CD3 and CD4 cell counts [59].

Sex chromosome constitution could also play an important role in disease outcome. Females have two copies of X chromosomes while males have one. The X chromosome contains a high density of immune-related genes. Numerous genes that are present on X chromosome include pattern recognition receptors (PRRs) such as *toll-like receptor 7* (*TLR7*), *TLR8*, and interleukin-1 receptor associated kinase 1 (*IRAK1*). Other genes include CXCR3, NFκB essential modulator (NEMO), GATA1, FOXP3, IL-2R γ chain, IL-3R α chain, and IL-13 α chain [52, 60]. TLRs’ expression has been found to be sex-specific as TLR3, TLR7, and TLR9 are female-biased while TLR2 and TLR4 are male-biased. TLRs can bind to pathogen-associated molecular patterns (PAMPs). TLR3, TLR7, and TLR9 have been shown to provide protection against virus by recognizing viral RNA and DNA while TLR2 and TLR4 provide protection against bacteria by recognizing the PAMPs on cell wall [54, 60]. This gene diversity in males and females may be one of the factors for modulating sex differences in immune responses to pathogens.

The early antiviral response by the innate sensing of SARS-CoV-2 genetic material by the PRR including TLR7 could be an important step [8]. Since TLR7 escapes X chromosome inactivation and is activated by estrogen [51], females may have a better strategy to overcome the early attack of SARS-CoV-2.

A study by Marquez et al. highlighted important sex differences in immune responses using human peripheral blood mononuclear cells (PBMCs). The authors showed that genomic differences between sexes increased after age 65. The innate and pro-inflammatory activity increased but adaptive immunity decreased in males [61]. Opposing effects were observed in B cells of males and females, with females showing activation of B cell-specific loci/genes but inactivation in males. The frequency of B cells also declined in older males [61]. In another study with a Japanese cohort, it was observed that the age-related decline of immune cells including B cells, T cells, and natural killer cells was slower in females compared to males. The rate of decline in IL-6 and IL-10 production was also slower in females [62]. This suggests that the immune system of women is well maintained for a longer time and that this may provide immune benefits to tolerate infections. Moreover, the stronger innate and adaptive immune response in females may help to clear the pathogens faster than in males. This could be a reason why females are showing less severity and mortality towards COVID-19.

The mortality rate due to SARS-CoV in 2002–2003 was high, and interestingly, this virus also showed higher mortality in males compared to females. Using SARS-CoV in the murine model, it was observed that the SARS-CoV can show high mortality in male mice [63]. Following SARS-CoV challenge, the levels of proinflammatory cytokine, interleukin-6 (IL-6), and chemokines including C-C motif chemokine ligand 2 and C-X-C motif chemokine ligand 1 (CCL2 and CXCL1) expression were elevated in the lungs of male mice compared to females. The authors suggested that this could lead to a prolonged inflammatory response in males and consequently lead to lung immunopathology and increased mortality [63].

In the case of SARS-CoV and MERS, increased level of proinflammatory cytokines was observed. Cytokines including IL-1B, IL-6, IL-12, IFNγ, putative internal head protein 10 (IP10), and monocyte chemoattractant protein 1 (MCP1) were increased in SARS-CoV patients [64] while IFNγ, TNFα, IL-15, and IL-17 were increased in MERS patients [65]. The immunopathology of COVID-19 is not fully understood, and most of the knowledge is based on SARS-CoV and MERS. Analysis of COVID-19 patients revealed that IL-2, IL-6, IL-7, IL-10, granulocyte stimulating factor, interferon γ inducible protein 10, monocyte chemoattractant protein 1, macrophage inflammatory protein 1-α, and tumor necrosis factor α were increased [2, 66, 67]. Among these immune system modulators, IL-6 and IL-10 showed a positive correlation with mild COVID-19 group while IL-6 showed a good correlation with severe cases [67]. Hence, blocking IL-6 activity to reduce COVID-19 severity has been proposed [66] and clinical trials are ongoing to analyze the efficacy of therapeutic antibodies against IL-6 (siltuximab) or IL-6 receptor (tocilizumab and sarilumab) [68].

Another important immunomodulator, high mobility group box 1 protein (HMGB1), could be a contributing factor for sex-specific severity and mortality from COVID-19. Infected or stressed cells usually release endogenous damage-associated molecular pattern molecules (DAMPs) that can activate the immune system by interacting with PRRs such as TLRs [69, 70]. Many DAMPs including biglycan, decorin, versican, fibrinogen, IL-33, defensin, DNA, RNA, F-actin, and HMGB1 have been identified [70]. Increased level of HMGB1 can induce proinflammatory cytokine (TNF, IL-1, and IL-6) release, and since chronic inflammatory diseases result in HMGB1 increase, the severity in COVID-19 patients with underlying inflammatory comorbidities could be correlated to HMGB1 levels [69]. Sex-related differences in immune responses can lead to differential mode of action to clear the viral load and damaged cells. Stressed pulmonary endothelial cells in males are known to undergo necrosis while in females apoptosis is more common [71]. Since necrosis releases more HMGB1 than apoptosis [71], this indicates that HMGB1 levels are differentially regulated in male and female COVID-19 patients with higher levels in males that further contributes to the severity and high mortality in males.

## Sex hormones and viral infection

Gonadal hormones not only are involved in the differentiation of reproductive organs, but also exert sex-specific regulation to multiple tissues including brain and those of the immune system [52, 72, 73]. In humans, sex chromosome constitution (XX/XY) determines sex and the *sex determining region Y* (*SRY*) gene present on Y chromosome is the master regulator gene of sex differentiation [74]. SRY drives testis differentiation by activating downstream genes, and the cascade of gene activation primes the gonad to secrete testosterone. The secreted testosterone further helps to differentiate the male reproductive systems. Testosterone is also suggested to reach the brain and organize neuronal networks. Testosterone can mediate gene expression directly by binding to the androgen receptor (AR) or indirectly following conversion into estrogen by the enzyme aromatase [73, 75]. On the other hand, estrogen and progesterone are important hormones in females that lead to differential regulation of reproductive and immune systems [51]. There are three different types of estrogens produced in females: estrone (E1), 17β-estradiol (E2), and estriol (E3). E2 is the predominant form that is produced by ovaries, and the level of hormones fluctuates during ovulation and pregnancy. Estrogens act through estrogen receptors (ERs) which exist in two forms, ERα and ERβ [76].

The expression of both ERα and ERβ has been identified in human immune cells, including B and T lymphocytes, mast cells, macrophages, dendritic cells, monocytes, and natural killer cells [77–79]. The expression of ERs have been shown to be cell specific as ERα was found to be the predominant form in CD4^+^ T cells, and ERβ was the predominant form in B cells [78]. Sex- and age-specific expression of ERα has been identified in human monocytes with higher expression in post-menopausal females and males than pre-menopausal females [78]. However, sex-dependent ER expression was not observed in B cells and T cells. Since there was no difference in ER expression in male and female T and B cells, the authors argued that the sex differences in immune response may not be a direct effect of estrogen but could be indirect through gonadotropin-releasing hormone [78].

Nakada et al. using a murine model showed that the ERα RNA levels were higher in male hematopoietic stem cells (HSCs) than in female HSCs and that the level of ERβ was low in both male and female HSCs. The authors also found that HSCs in females divide more frequently than in males. Interestingly, conditional deletion of ERα resulted in reduced HSC proliferation in females but not in males [80].

Males and females are under the influence of different hormonal milieu. Testosterone has been shown to have immunosuppressive effect while estrogen has immunoenhancing effect [60]. Testosterone has been found to inhibit T helper cell differentiation [81] and positively correlate with the viral load of Venezuelan equine encephalitis virus in macaques [82]. Testosterone is also known to decrease the responsiveness towards influenza vaccine [83]. Analysis of testosterone levels in females showed that the level is usually low in females suffering from autoimmune disease compared to healthy females [55]. Furthermore, androgen ablation in male mice showed improved performance of immune cells towards prostate cancer [84]. Androgen ablation also alters immune organs as the thymus and spleen have been reported to increase weight [85]. Although the mature peripheral CD4 T cell population declined, the one that reached the spleen showed enhanced activation [85]. This suggests that male sex hormones (androgens) could lead to susceptibility and severity towards pathogenic infections. It is indicated that prostate cancer patients who are under androgen-deprivation therapy (ADT) to regulate androgen production have lowered risk for SARS-CoV-2 infection compared with patients who did not receive ADT [86]. The authors argued that [86] since TMPRSS2 expression is induced by androgens [87], the ADT could downregulate TMPRSS2 and this in turn could be lowering SARS-CoV-2 infection. It is suggested that ADT could be beneficial for COVID-19, and since this disease progresses rapidly, the ADT intervention will be beneficial during the initial stage of viral infection and not in later stages [88]. However, the expression of TMPRSS2 in human male and female lungs is not different [89, 90], and in mice models, treatment with enzalutamide, an AR antagonist, did not result in decreased pulmonary TMPRSS2 expression [89]. Hence, the use of AR antagonists to regulate TMPRSS2 expression for COVID-19 warrants further research.

Estrogen, on the other hand, provides protection against pathogens as it has antiviral properties in different viral infections including HIV, hepatitis C virus, Ebola, and human cytomegalovirus [91]. Estrogen was shown to inhibit influenza A virus replication in cultured nasal epithelial cells isolated from female mice [91]. Interestingly, the protective effect of estrogen was not observed in nasal epithelial cells isolated from male mice [91]. Since SARS predominantly replicates in airways, higher estrogen levels in females may increase the protection from SARS infections [63]. Inhibition of ER function using ER antagonist ICI 182,780 resulted in higher SARS-CoV infections in females. However, gonadectomy or treatment with the AR antagonist flutamide did not affect morbidity or mortality in male mice following SARS-CoV infection [63]. Based on this, it was suggested that the estrogen receptor signaling plays an important role in coronavirus infection and mortality, while androgens do not play a role in pathogenesis [63]. This suggests that estrogen signaling is critical in regulating viral infection and could be one of the reasons why females are showing fast recovery and low mortality from COVID-19.

## Testis and viral clearance

The testis is an immune-privileged organ as both allo- and auto-antigens are incapable of provoking immune response. This characteristic is important for keeping the immunogenic germ cells away from the immune response [92]. The unchecked immune system can react with the surface antigen on sperm cells known as meiotic germ cell antigen (MGCA), and this can result in infertility [92]. Although testis is an immune-privileged organ, it can elicit innate immunity when microbial pathogens infiltrate the organ. Viruses including HIV, cytomegalovirus, and mumps are known to infect testis and lead to testicular disorders [93]. Moreover, viruses including Zika, Ebola, and Marburg have been isolated from semen samples and they are known to be transmitted sexually [94].

Shastri and coworkers showed that males in families required a longer time to recover from COVID-19 than other family members. The authors observed that testis had a high expression of ACE2 both at the mRNA and protein levels [26]. The authors suggested that coronavirus can enter the testis and consequently lead to higher viral load and take more time for viral clearance. The total subjects in this study was only 68 (48 males and 20 females) with a median age of 37 years. A thorough study with a higher number of patients is required to support the author’s hypothesis.

**Fig. 4:**
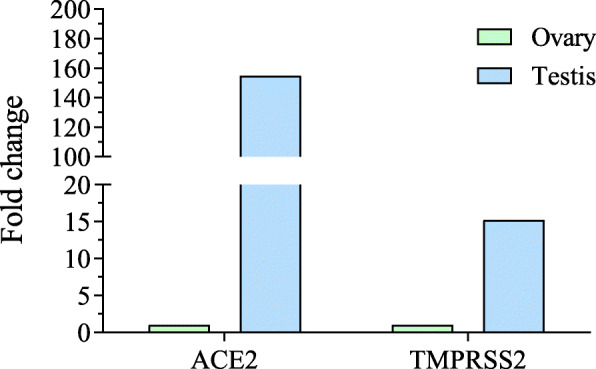
Differential expression of ACE2 and TMPRSS2. The RNA sequencing data was obtained from the NCBI database (Bioproject, PRJEB4337) submitted by a previous study [96]. The raw reads were aligned to the human genome, and differential gene expression was determined using Partek Flow Software. The parameters were as follows: *p* value 0.05, false discovery rate 0.05, and fold change ± 2

In another study, coronavirus genetic material was detected in the semen samples of males infected with coronavirus [95]. Analysis of RNA sequencing data obtained from the NCBI database [96] showed that both *ACE2* and *TMPRSS2* were highly expressed in the testis compared to the ovary (Fig. 4). This supports the observation that the coronavirus may enter the human testis. The sex hormones in males were also altered following infection with SARS-CoV-2. Analysis of 81 infected men with SARS-CoV-2 showed increased luteinizing hormone (LH), but the ratio of testosterone to LH and follicle stimulating hormone to LH was decreased [97]. Although coronavirus RNA was detected in semen samples, active viral particles have not been isolated from testis so far. Hence, the theory of involvement of testis in delayed viral clearance and high mortality in men [26] should be taken with caution.

Disturbance of immune regulation in testis can result in orchitis, a condition where leukocytes infiltrate testis and damage seminiferous tubules and consequently result in infertility. Hence, whether testis is a contributing factor in poor prognosis and high mortality in men needs to be accessed more thoroughly. In the SARS outbreak in 2002, higher mortality was observed in males compared to females. The infected individuals showed multiple organ damage, and in males, testis was also affected with germ cell destruction, lack of spermatozoon, thickened basement membrane, and leukocyte infiltration [98]. However, no viral particles or viral RNA was detected in the tissue samples and it was suggested that the virus-mediated testis damage was due to an immune response.

## Conclusions and perspective

The effects of COVID-19 pandemic will be experienced for a long time as not only it has resulted in high mortality but also it will have a huge impact on health as well as the economy. The world has already experienced three coronavirus outbreaks in the past 20 years (SARS-CoV, 2002; MERS, 2012; and SARS-CoV-2, 2019), and the source of these viruses was animals. Bats have been identified as a reservoir of many viruses including SARS-CoV, MERS, Ebola, Rabies, Nipah, and Hendra [99]. There is a wide variety of bat species, and they offer a good breeding ground for viruses [99]. This suggests that the outbreak so far could be just the tip of an iceberg and that the zoonotic transfers must be carefully monitored.

**Fig. 5:**
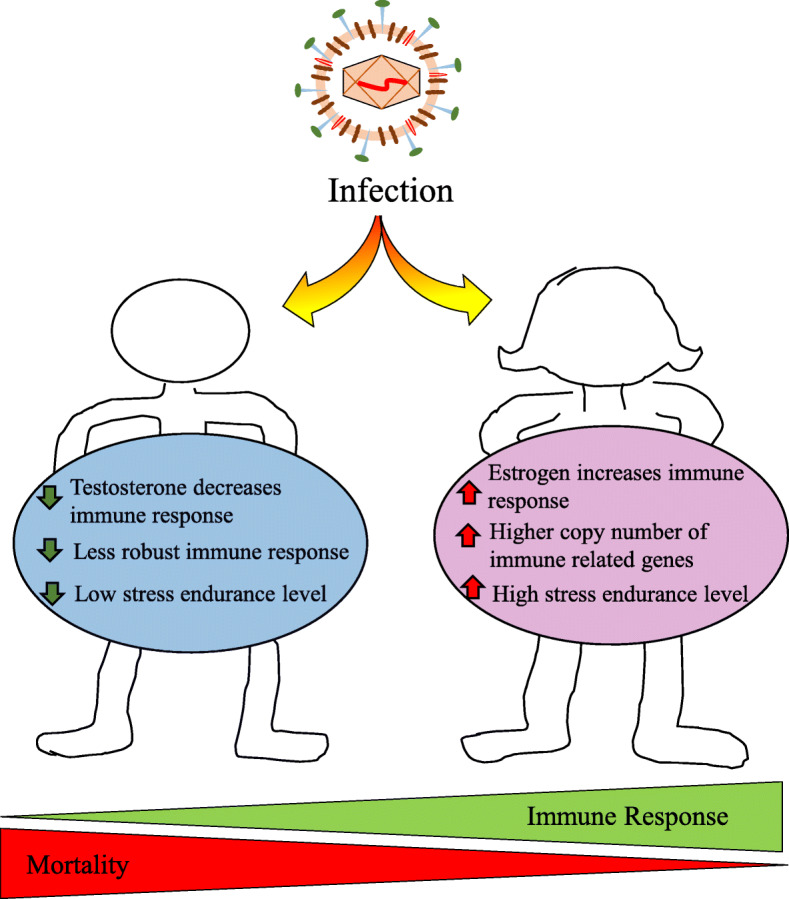
The possible factors in sex-biased mortality from COVID-19. COVID-19 has shown sex-biased mortality with higher death rate in males. This difference could be due to testosterone and estrogen differential effect on the immune system. Since females have two X chromosome, they have higher copy number of immune-related genes which provides a stronger immune response. The robust immune system in females better regulates viral infections, and consequently, lower mortality is observed. The stress endurance level is low in males compared with females, and this could also be a contributing factor for the sex-specific pathogenesis from COVID-19

COVID-19 has shown a clear male-biased severity and mortality in different countries. The sex-biased pathogenesis is not understood properly, but it could be multifactorial. The difference in immune system function between males and females could be an important determinant. Females are known to show a robust immune response to pathogens which could help them to better regulate viral load and viral clearance compared with males. Since many immune genes are present on X chromosome, the XX and XY genetic constitutions could also contribute to COVID-19 severity. Other differences including steroid hormone milieu and sex organs could also play a crucial role in pathogenesis (Fig. 5). Estrogen in females can have immune-enhancing effects while testosterone secreted by the testis can have immune-suppressive effects. However, there is no sufficient clinical data to show that the SARS-CoV-2 can enter the testis and regulate COVID-19 severity and mortality in males. Hence, testis involvement should be further explored to understand male-biased mortality. The stress endurance level in males and females is also different with females showing higher endurance against different stress including food shortage and pathogens. This could also be a contributing factor in sex-biased pathogenesis from COVID-19.

The sex-biased mortality from COVID-19 suggests that research should be focused on identifying sex-related differences associated with physiology, immune function, and developmental processes. Fewer female animals are used in research and clinical trials and that the preferred choice of models remains males [100, 101]. The inclusion of both males and females in research is needed to bridge the gap of drug discovery, drug toxicity, and pathogenesis.

## Data Availability

Not applicable

## Abbreviations

ACEI: Angiotensin-converting enzyme inhibitor
ACE2: Angiotensin-converting enzyme 2
ADT: Androgen-deprivation therapy
AR: Androgen receptor
ARB: Angiotensin II type 1 receptor blocker
ER: Estrogen receptor
COVID-19: Coronavirus disease 19
DAMP: Damage-associated molecular pattern molecule
HMGB1: High mobility group box 1 protein
HSC: Hematopoietic stem cell
NTD: N-terminal domain
MERS: Middle East respiratory syndrome
MGCA: Meiotic germ cell antigen
RBD: Receptor binding domain
SARS: Severe acute respiratory syndrome coronavirus
SRY: Sex determining region Y
TMPRSS2: Transmembrane protease/serine subfamily member 2
